# Quantification of biventricular myocardial function using cardiac magnetic resonance feature tracking, endocardial border delineation and echocardiographic speckle tracking in patients with repaired tetralogy of fallot and healthy controls

**DOI:** 10.1186/1532-429X-14-32

**Published:** 2012-05-31

**Authors:** Aleksander Kempny, Rodrigo Fernández-Jiménez, Stefan Orwat, Pia Schuler, Alexander C Bunck, David Maintz, Helmut Baumgartner, Gerhard-Paul Diller

**Affiliations:** 1 Adult Congenital and Valvular Heart Disease Center, Department of Cardiology and Angiology, University Hospital of Muenster, Albert-Schweitzer-Str. 33,, 48149, Münster, Germany; 2 Coronary Care Unit, Cardiovascular Institute, Hosp Clinico San Carlos, Madrid, Spain; 3 Department of Clinical Radiology, University Hospital of Muenster, Muenster, Germany

## Abstract

**Background:**

Parameters of myocardial deformation have been suggested to be superior to conventional measures of ventricular function in patients with tetralogy of Fallot (ToF), but have required non-routine, tagged cardiovascular magnetic resonance (CMR) techniques. We assessed biventricular myocardial function using CMR cine-based feature tracking (FT) and compared it to speckle tracking echocardiography (STE) and to simple endocardial border delineation (EBD). In addition, the relation between parameters of myocardial deformation and clinical parameters was assessed.

**Methods:**

Overall, 28 consecutive adult patients with repaired ToF (age 40.4 ± 13.3 years) underwent standard steady-state-free precession sequence CMR, echocardiography, and cardiopulmonary exercise testing. In addition, 25 healthy subjects served as controls. Myocardial deformation was assessed by CMR based FT (TomTec Diogenes software), CMR based EBD (using custom written software) and STE (TomTec Cardiac Performance Analysis software).

**Results:**

Feature tracking was feasible in all subjects. A close agreement was found between measures of global left (LV) and right ventricular (RV) global strain. Interobserver agreement for FT and STE was similar for longitudinal LV global strain, but FT showed better inter-observer reproducibility than STE for circumferential or radial LV and longitudinal RV global strain. Reproducibility of regional strain on FT was, however, poor. The relative systolic length change of the endocardial border measured by EBD yielded similar results to FT global strain. Clinically, biventricular longitudinal strain on FT was reduced compared to controls (P < 0.0001) and was related to the number of previous cardiac operations. In addition, FT derived RV strain was related to exercise capacity and VE/VCO_2_-slope.

**Conclusions:**

Although neither the inter-study reproducibility nor accuracy of FT software were investigated, and its inter-observer reproducibility for regional strain calculation was poor, its calculations of global systolic strain showed similar or better inter-oberver reproducibility than those by STE, and could be applied across RV image regions inaccessible to echo. ‘Global strain’ calculated by EBD gave similar results to FT. Measurements made using FT related to exercise tolerance in ToF patients suggesting that the approach could have clinical relevance and deserves further study.

## Background

Tetralogy of Fallot (ToF) is the most common cyanotic heart defect at birth with a reported incidence of 3.9 per 10,000 live births [1]. In the current era, most patients undergo corrective surgery in early life and long-term survival prospects are excellent. However, long-term complications such as pulmonary valve regurgitation, right (RV) and left ventricular (LV) dysfunction and the risk for malignant arrhythmias and sudden cardiac death remain [2,3]. Assessment of the function and size of the LV and RV is, thus, essential in the follow-up and treatment of ToF patients [4,5]. Echocardiography remains the main imaging modality in the routine follow-up of these patients. However, due to the complicated geometry of the tripartite RV and limited acoustic windows in many patients, conventional echocardiography is not well suited for assessing RV volumes and RV function [6-8]. These measurements remain the domain of cardiac magnetic resonance imaging (CMR), offering the advantages of a wide field of view, lack of anatomic plane restriction, and superior reproducibility [9,10]. Beyond RV enlargement and dysfunction, LV dysfunction is not uncommon in ToF patients and has been related to increased risk of sudden cardiac death in this setting [11-14]. Although CMR has a superior interstudy reproducibility of LV volumes, ejection fraction and mass measurements compared to echocardiography, we have recently demonstrated that parameters of LV longitudinal function [15], as assessed by speckle tracking echocardiography are more sensitive compared to ejection fraction in detecting subtle LV systolic dysfunction and in predicting prognosis in this setting [16,17]. Similarly, assessing parameters of RV longitudinal function is theoretically appealing as anatomical studies have demonstrated that the deeper RV muscle fibres are predominantly arranged in a longitudinal fashion from the tricuspid valve annulus to the apex and RV stroke volume grossly depends on longitudinal shortening [18,19].

Until recently, measurement of LV and RV longitudinal function has been the province of echocardiography, with methods such as M-mode, tissue Doppler and more recently speckle tracking echocardiography. While tissue tagging CMR or harmonic phase (HARP) analysis could offer an alternative to echocardiography they require additional sequence acquisition, special expertise and are not widely available [20].

Recently, however, CMR feature tracking (FT) software designed to derive myocardial strain and strain rate from standard CMR cine images has become available [21]. Its image post processing is comparable to, but not the same as, that of speckle tracking echocardiography (STE), aiming to provide multi-planar strain data from steady-state-free precession (SSFP) cine CMR images without the need for tagged images [22-25]. In this study, we set out to compare FT measurements of strain with results obtained by STE, to assess the inter-observer reproducibility of global and regional strain calculations by FT, and to evaluate the relation of FT derived parameters to the clinical status in adult patients with ToF. In addition, we hypothesised that comparable measurements of global strain might be obtained by a simple algorithm assessing the relative systolic length change of the endocardial-blood border through systole.

## Methods

We included 28 consecutive adult patients (age ≥ 18 years) with repaired ToF that underwent CMR and TTE at the Adult Congenital and Valvular Heart Disease Center at the University of Muenster/Germany. In addition, 25 individuals without cardiovascular disease served as controls. Patients and controls underwent clinical examination, transthoracic echocardiography, CMR and cardiopulmonary exercise testing. This study was approved by the local Ethics Committee.

### Speckle tracking based myocardial deformation analysis

Transthoracic echocardiography was performed according to the current guidelines as described in detail previously [16,26-29]. Briefly, it was performed in all subjects with a Vivid 7 Dimension system (Vingmed, General Electric, Milwaukee, Wisconsin). All recordings were stored digitally in DICOM format for offline analysis. The cine loops for assessment of peak longitudinal 2D strain of the left and right ventricle were recorded in apical 4-chamber views and optimized through changing the transducer scan width to achieve a frame rate of at least 40 per second. Echocardiographic data were exported to TomTec Image Arena. All echocardiographic measurements were performed using TomTec software (Myocardial Performance Analysis package). The echocardiographic speckle-tracking analysis was performed separately for the RV and LV and consisted of marking the endocardium, defining the width of the region of interest and performing the automatic computation. Peak longitudinal strain for both ventricles was measured in the apical four chamber view. Circumferential and radial strain of the left ventricle (LV) were measured in the parasternal short axis view on the level of papillary muscles. The assessment of circumferential and radial deformation parameters of the right ventricle (RV) was not feasible in nearly all subjects on echocardiography due to suboptimal acoustic windows and was, therefore, not included into the statistical analysis.

### Feature tracking based on CMR

All CMR scans were performed on a 1.5-Tesla system (Achieva, Philips, Best, The Netherlands) using a five-element phased array cardiac synergy coil and with image acquisition and subsequent analysis according to current guidelines [28].

For cine imaging a single-slice two-dimensional (2D) balanced steady-state free precession (b-SSFP) sequence in breath-hold technique and with retrospective ECG triggering was used. Imaging parameters were chosen as follows: echo time (TE) and repetition time (TR) were set to shortest resulting in an average TR of around 4 ms and a TE of 2 ms slightly varying with slice orientation; typically 25 phases per cardiac cycle; reconstructed in-plane resolution 1 mm; slice thickness 6 mm for axial planes and 8 mm for short axis planes. The typical temporal resolution of the cine b-SSFP sequences was 30 to 40 ms depending on the heart rate. The total time for cine imaging was 15 to 20 minutes. The sequences were exported in DICOM-Format without special adjustments.

**Figure 1:**
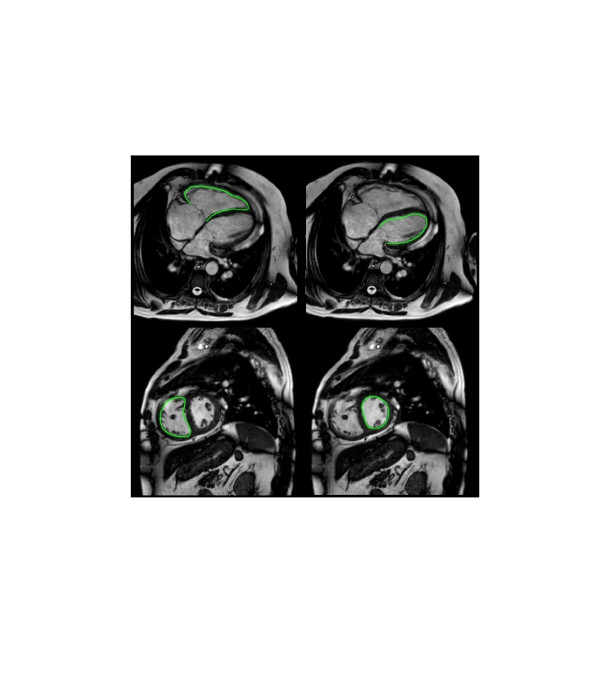
Example of feature tracking based assessment of the right and left ventricle on CMR using feature tracking (TomTec Diogenes software).

FT analysis was performed using the recently introduced TomTec Diogenes software (Additional file 1, Version 1.1.0.2). It was done separately for LV and RV and consisted of marking the endocardium and triggering the automatic computation (Figure 1, Additional files 1,2,3 and 4). As in STE the consistency of the movement of myocardium and the tracking contour was assessed visually and the tracking analysis was repeated if needed. Longitudinal deformation parameters of the LV and RV were assessed in a four chamber view. Circumferential and radial parameters of both LV and RV were assessed in the short axis view at the level of the papillary muscles. In order to assess interobserver agreement both STE and FT were performed by two operators blinded to each others results (AK and GPD). The details of the FT algorithm have been published previously [22,23]. Feature tracking is based on an algorithm for endocardial border tracking (i.e. 1-dimensional tracking perpendicular to the endocardial border), supplemented by 2-dimensional tracking of myocardial segments capitalizing on inhomogeneity of tissue brightness, anatomical features (such as papillary muscles or trabeculations) and “roughness” of the cavity-myocardial border using a maximum likelihood method. In addition, smoothing filters are applied to adjacent tracking points to improve spatial coherence. The technique is different from border detection as it relies on an initial border delineation drawn manually by the operator on a single frame. The software algorithm then follows this border throughout the cardiac cycle automatically.

### Endocardial border delineation on CMR

The length of the endocardial border throughout the cardiac cycle was measured using custom written software code (MatLab Version R2011b Software and Image Processing Toolbox, MathWorks, Inc., Natick, MA, USA). The software code imports a CMR sequence in DICOM format and allows the user to delineate the endocardial border manually by defining points on the border for each frame. Additional points are generated by (a piecewise cubic Hermite) interpolation. The length of the border is determined as the sum of Euclidean distances between points and the border length is plotted to allow the user to determine maximal and minimal border length. Figure 1 illustrates the analysis. Based on these readings global systolic strain (ε) is determined in accordance to a general definition of strain as:

(1) $$\epsilon =100\%\cdot \frac{L-L_{0}}{L_{0}}$$

with *L*_*0*_ as the length before deformation and *L* as the length after deformation.

This global strain is only dependent on the overall border length and does not require to accurately track individual myocardial segments 2-dimensionally. The MatLab software code is available upon request from the authors for academic purposes. All global strain values are reported as absolute values.

### Cardiopulmonary exercise testing

Cardiopulmonary exercise testing (CPET) was performed on an upright bicycle ergometer as described in detail previously [16]. Subjects were encouraged to exercise to exhaustion. Ventilation, oxygen uptake, and carbon dioxide production were measured continuously (Jaeger SBx/CPX MS-CPX, Hoechberg, Germany). Heart rate was assessed by continuous electrocardiography (QRS-Card/232, VIASYS Healthcare) and arterial blood pressure was recorded manually by sphygmomanometry. Peak power (in Watts) was also recorded. The achieved values were referred to the reference values [30].

### Statistical analysis

Data are presented as mean ± standard deviation or median and interquartile range (IQR), depending on data distribution. All strain data are presented as absolute values. Data was assessed for normal distribution using the D'Agostino-Pearson test. In case of normal distribution comparison between two groups was performed using a two-tailed *t*-test and Welch-test in case of unequal variances (depending on the results of an F-test). Categorical variables were compared using chi-square test. For all analyses, a *P*-value < 0.05 was considered statistically significant. Correlation coefficients were calculated using Pearson or Spearman formula depending on data distribution. Agreement between STE and FT was assessed using coefficients of variability (COV) [10,31]. In addition, Bland Altman plots are provided [32]. Statistical analyses were performed using MedCalc for Windows, version 11.6.1.0 (MedCalc Software, Mariakerke, Belgium) and graphs were prepared using R-package version 2.13.0.

## Results

Demographic and conventional CMR data of patients and controls are presented in Table 1.

**Table 1:** Demographic parameters and basic volumetric data in patients with tetralogy of Fallot and controls

		**Patients (n = 28)**	**Controls (n = 25)**	***P*-Value**
Age	yrs.	40.4 ± 13.3	33.1 ± 15.7	0.07
Male/female	n	15/13	15/10	0.85
Age at ToF repair	yrs.	5.7 (IQR 4.5 - 7.5)	-	-
Cardiac surgeries (1/2/3)	n	17/8/3	-	-
Weight	kg	75.2 ± 14.9	70.1 ± 11.2	P = 0.22
Height	cm	173.0 ± 9.2	176.8 ± 8.9	0.18
Indexed LV EDV	ml/m^2^	80.7 ± 22.5	78.8 ± 11.2	0.76
Indexed LV ESV	ml/m^2^	36.4 ± 16.8	27.5 ± 5.1	**0.04**
LV EF	%	56.5 ± 9.7	63.6 ± 5.7	**0.01**
Indexed RV EDV	ml/m^2^	133.5 ± 48.4	77.7 ± 9.9	**0.0001**
Indexed RV ESV	ml/m^2^	75.2 ± 35.7	34.7 ± 10.0	**0.0002**
RV EF	%	45.8 ± 11.2	55.3 ± 6.6	**0.01**
PVR (mild/moderate/severe)	n	11/5/12	-	-

*ToF* tetralogy of Fallot*, BSA* body surface area*, BMI* body mass index*, LV EDV* left ventricular end diastolic volume*, LV EF* left ventricular ejection fraction*, LV ESV* left ventricular end systolic volume*, PVR* pulmonary valve regurgitation*, RV EDV* right ventricular end diastolic volume*, RV EF* right ventricular ejection fraction*, RV ESV* right ventricular end systolic volume. All volumetric data are based on cardiac magnetic resonance imaging.

### Feasibility of feature tracking CMR

FT measurements could be performed for the LV and RV in all ToF patients included in the study. One complete analysis, including package creation for the short axis and four chamber view and analysis of RV and LV longitudinal, radial and circumferential strain required approximately 5 minutes (305 ± 23 seconds).

### Comparison of FT and STE derived data

**Figure 2:**
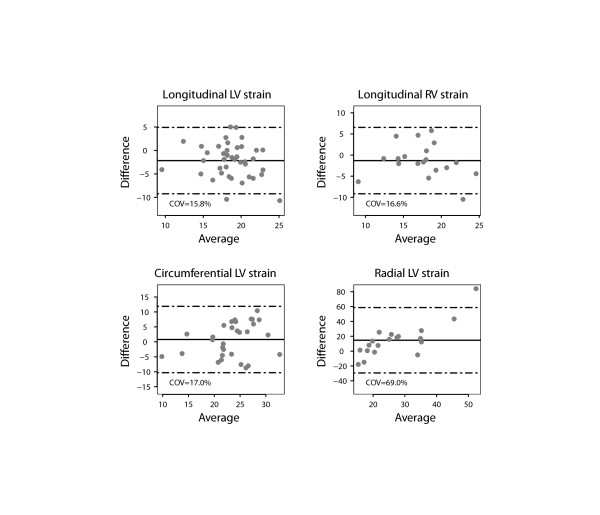
**Bland Altman plot demonstrating the agreement for global strain values of the left (LV) and right ventricle (RV) measured using speckle tracking echocardiography (STE) and feature tracking (FT).** Horizontal solid line represents the mean difference (STE-FT) and the dashed lines mean +/− 2 standard deviations of the difference.

**Figure 3:**
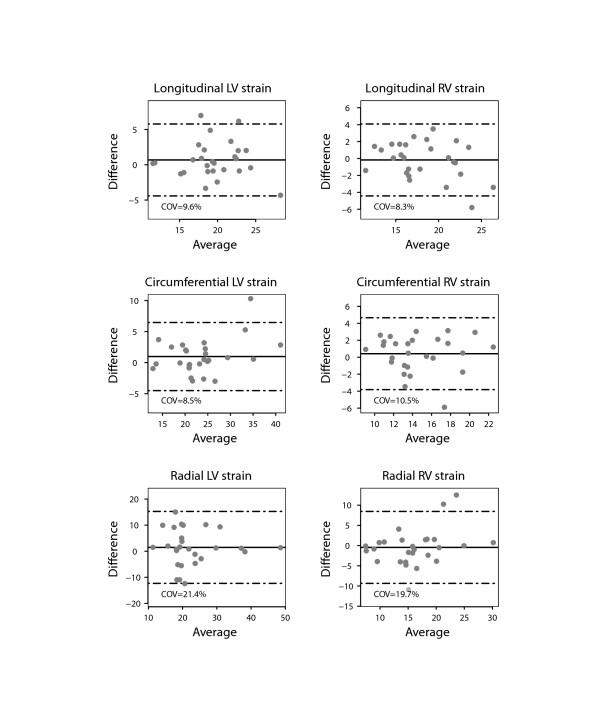
**Bland Altman plot demonstrating interobserver agreement for global strain values of the left (LV) and right ventricle (RV) measured using feature tracking (FT) on CMR.** Horizontal solid line represents the mean difference and the dashed lines mean +/− 2 standard deviations of the difference.

**Figure 4:**
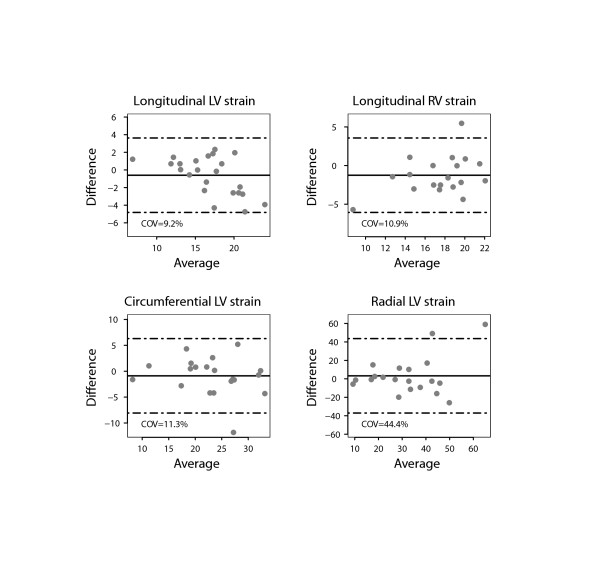
**Bland Altman plot demonstrating interobserver agreement for global strain values of the left (LV) and right ventricle (RV) measured using speckle tracking echocardiography (STE)**. Horizontal solid line represents the mean difference and the dashed lines mean +/− 2 standard deviations of the difference.

**Figure 5:**
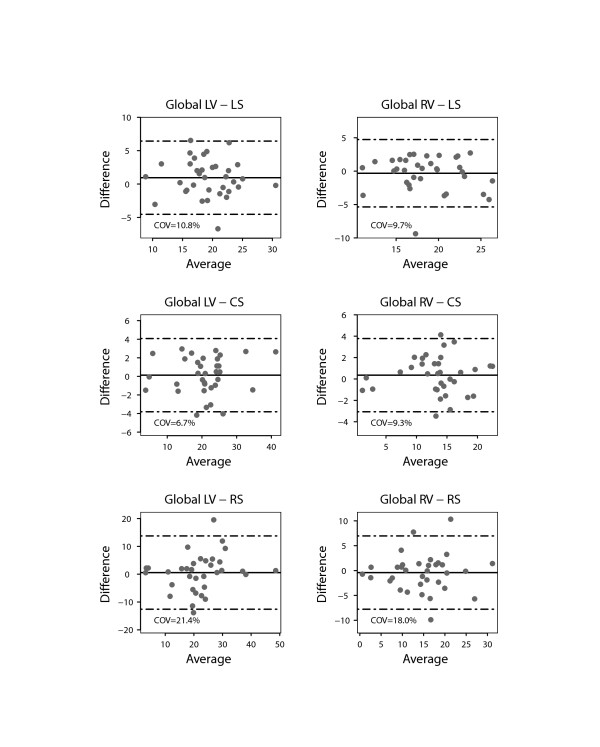
**Bland Altman plot demonstrating intraobserver agreement for global strain values of the left (LV) and right ventricle (RV) measured using feature tracking (FT) on CMR**. Horizontal solid line represents the mean difference and the dashed lines mean +/− 2 standard deviations of the difference.

**Figure 6:**
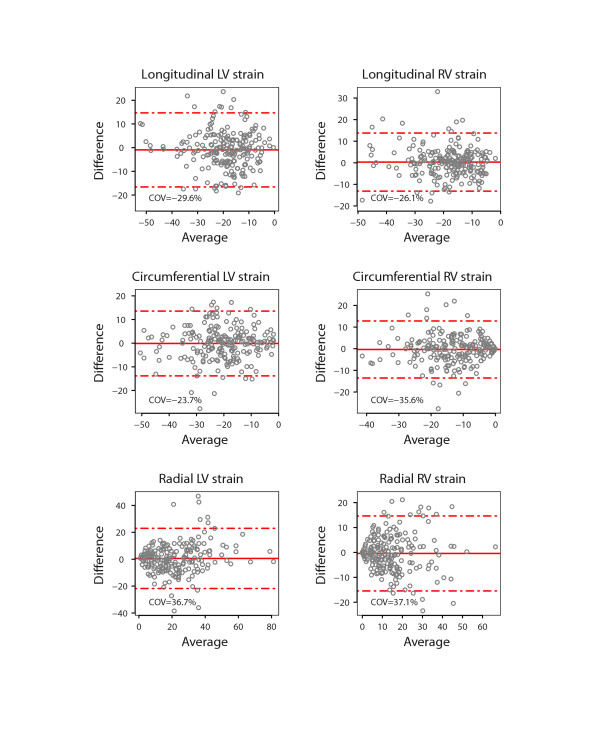
**Bland Altman plot demonstrating intraobserver agreement for regional strain values of the left (LV) and right ventricle (RV) measured using feature tracking (FT) on CMR.** Horizontal solid line represents the mean difference and the dashed lines mean +/− 2 standard deviations of the difference.

There was a close agreement between longitudinal and circumferential global LV strain derived by STE and FT and poor agreement for radial LV global strain (Figure 2). There was also a good agreement between the RV longitudinal global strain measurements on FT and STE. Figures 3 and 4 show the interobserver agreement for global strains on FT and STE respectively. It was found to be comparable for longitudinal LV strain on FT and STE (COV = 9.6%/9.2%, respectively). In contrast, the interobserver agreement was found to be better on FT compared to STE for circumferential and especially radial LV global strain (COV = 8.5%/11.3% for CS, COV = 21.4%/44.4% for RS). In addition, interobserver agreement for the longitudinal RV global strain was superior for FT compared to STE (COV = 8.3%/10.9%). The intraobserver agreement for global strain was found to be comparable for all strain measurements to the interobserver agreement (Figure 5). In contrast to global strain, reproducibility of segmental strain on FT was poor with COV ranging between 23.7% and 37.1% as illustrated in Figure 6.

### Comparison of FT and EBM derived data

**Figure 7:**
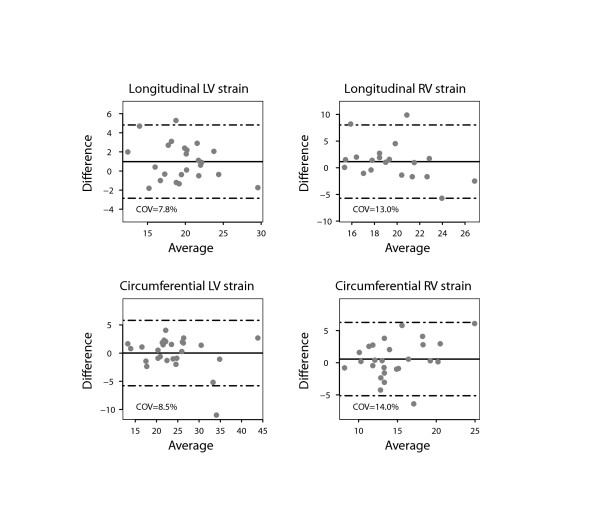
***Bland*Altman plot demonstrating the agreement for global strain values of the left (LV) and right ventricle (RV) measured using endocardial border delineation (EBD) and feature tracking (FT).** Horizontal solid line represents the mean difference (EBD-FT) and the dashed lines mean +/− 2 standard deviations of the difference.

**Figure 8:**
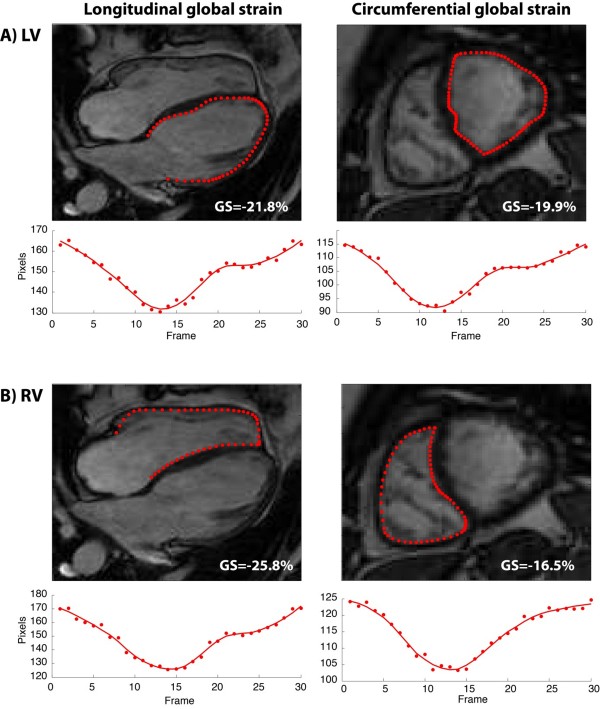
**Example of endocardial border delineation based assessment of the right and left ventricle on CMR using custom written software.** GS=global strain.

Figure 7 shows Bland Altman plots for the comparison between FT and EBM derived data in patients with ToF. It shows a good agreement between the methods with values of COV between 7.8% and 14%. Due to the nature of EBM this comparison can only be performed for global strain (Figure 8).

### LV size and function

**Figure 9:**
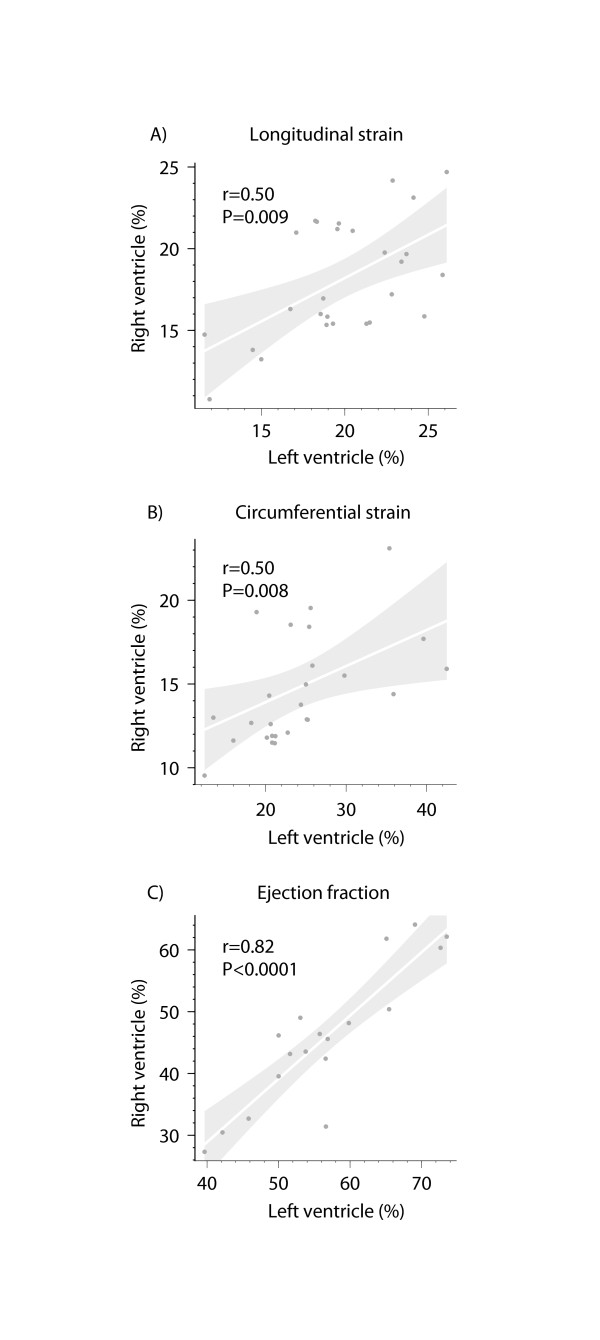
**Correlations between right and left ventricular A) longitudinal and B) circumferential global strain on FT as well as C) ejection fraction inpatients with Tetralogy of Fallot and controls illustrating the presence of interventricular interaction.** P-values refer to Spearman rank test. Grey area represents values within 95% confidence interval for the regression line (white).

There was no significant difference in indexed end-diastolic LV volume and ejection fraction between patients and controls (Table 1). On FT there was also no significant difference in LV circumferential strain (23.5 ± 6.0 vs. 22.0 ± 3.9%, *P* = 0.28). In contrast, longitudinal LV strain (19.2 ± 4.0 vs. 21.3 ± 3.3%, P = 0.048) and radial LV strain (22.0 ± 8.9 vs. 28.0 ± 11.3, P = 0.2) were found to be lower in patients compared to controls. In addition, a significant interaction between LV and RV function in terms of ejection fraction, longitudinal and circumferential strain was found as illustrated in Figure 9.

### RV size and function

Indexed right ventricular enddiastolic (EDV) and endsystolic volumes (ESV) were found to be significantly higher in patients compared to controls (p < 0.001 for both, Table 1).

RV ejection fraction was significantly reduced in patients compared to controls (P = 0.01, Table 1).

**Figure 10:**
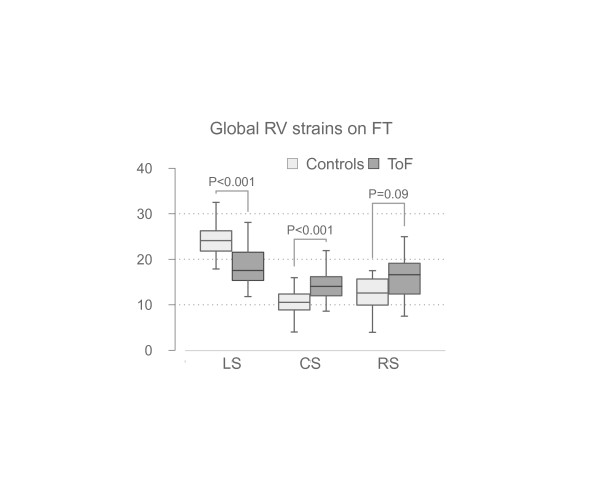
Global longitudinal (LS), circumferential (CS) and radial strain (RS) of the right ventricle (RV) derived from feature tracking (FT) in patients with Tetralogy of Fallot and controls.

On FT peak longitudinal RV strain was found to be reduced compared to controls (18.3 ± 4.3 vs. 24.1 ± 4.0%, *P* < 0.0001, respectively, Figure 10). It was also correlated with the number of previous cardiac operations (r = 0.67, P < 0.0001) as was RV ejection fraction (r = −0.52, P = 0.005). In contrast, RV circumferential strain was significantly higher in patients compared to controls (14.4 ± 3.6 vs. 10.6 ± 3.1%, *P* = 0.0002).

RV EF was significantly correlated with longitudinal (r = 0.51, P = 0.005) but not with circumferential (r = −0.12, P = 0.5) and radial strain (r = 0.04, P = 0.8).

### Exercise capacity and its relation to CMR parameters

Exercise capacity was significantly reduced in patients compared to published normal values (peak oxygen uptake of 19.2 ± 7.5 mL/kg/min = 62 ± 4% of the predicted value, P < 0.0001).

Peak oxygen uptake (PVO_2_) and VE/VCO_2_ slope were not related to the enddiastolic and endsystolic volume of LV and RV. There was a significant difference in VE/VCO_2_ slope on cardiopulmonary exercise test between the patients with and without severe pulmonary regurgitation (33.0 ± 5.7 vs. 26.7 ± 4.5, P = 0.008) but no significant difference in PVO_2_ (19.8 ± 8.1 vs. 18.6 ± 7.3, P = 0.7).

In contrast, several FT derived parameters of the RV, but not LV function, were related to exercise capacity in ToF Patients. Peak oxygen uptake correlated with RV radial strain (r = 0.49, P = 0.02), while VE/VCO_2_ slope correlated with RV radial and circumferential strain (r = −0.54, P = 0.01 and r = −0.56, P = 0.008, respectively). There was also a significant correlation of radial and longitudinal RV strain with peak power achieved on exercise (r = 0.47, *P* = 0.004 and r = 0.36, *P* = 0.03 respectively). Interestingly, there was no association of RV ejection fraction with any parameter of exercise tolerance.

## Discussion

The current study demonstrates that global LV and RV strain can be quantified using standard CMR sequences and there is a good agreement between FT derived global strain and strain measured on conventional STE. The repeated measurements performed with FT however showed, for most parameters, better interobserver agreement for FT compared to STE. The reproducibility was particularly good for circumferential LV and RV strain and longitudinal RV global strain and was comparable with previously reported interobserver agreement for RV volume and ejection fraction measurements by CMR [10]. In contrast to STE, FT also allowed measurement of RV strain - including circumferential and radial strain - in all subjects.

Patients late after correction of ToF are at risk for malignant arrhythmias and sudden cardiac death [33]. Risk stratification in this population, however, remains challenging.

Previous studies have suggested surgical history, ECG parameters, inducible arrhythmia, exercise intolerance and RV burden of myocardial fibrosis may carry prognostic information in this setting [3,33]. More recent studies, have highlighted the prognostic value of LV systolic dysfunction [14,33]. Although LV impairment is not uncommon in ToF patients, only a minority of patients present with more than mildly reduced LV ejection fraction [11,12]. As a consequence, more sensitive parameters of early LV dysfunction may be required. Recent studies in various cardiovascular conditions have demonstrated that measures of LV longitudinal function may more sensitive in detecting early myocardial damage than ejection fraction [16,34]. Using tagged CMR, Ordovas, et al. have demonstrated that early regional LV dysfunction is present in patients with preserved LVEF after repair of tetralogy of Fallot [35]. This illustrates the growing interest in assessing myocardial deformation in this challenging patient population using CMR techniques. The current study demonstrates that global systolic strain can be measured accurately using FT and standard SSFP sequences. Unlike earlier studies - concentrating entirely on short axis function - we show that this technology can also be applied to assess longitudinal LV and RV function. Whereas, a good intra- and interobserver agreement was found for measures of global strain, reproducibility of segmental strain on FT was poor in our hands as illustrated by Figure 6.

Details on the FT algorithm have been published [22,23]. In our interpretation, the accuracy of this technique in determining global strain mainly depends on the ability to accurately track the endocardial border, while evaluating segmental strain requires robust 2-dimensional tissue tracking. Based on our experience with the software and on previous results it appears that 1-dimensional-border tracking is accurate. The poor reproducibility of segmental strain may however highlight problems with 2D-segmental tracking in this setting [23]. It appears that this aspect of the algorithm requires further study before it should be applied routinely. This is supported by the lack of published data comparing, let alone validating, FT measurements of regional strain or longitudinal motion with a reference method such as CMT tagging .

Theoretically, global endocardial strain could be calculated from the change of length of the myocardial-blood border through the cardiac cycle. To test this concept, we used in-house software allowing the user to delineate the endocardial border throughout the cardiac cycle. Using this technique, the current study demonstrates a good agreement between EBM strain measurements with those obtained by FT (Figure 7). This approach (despite also requiring external validation) could emerge as a low cost alternative to FT for academic purposes or to measure strain in anatomic segments not currently supported by the TomTec software (e.g. within the RV outflow tract). The TomTec software, in contrast, has the advantages of (1) a timesaving algorithm to track the endocardial border automatically throughout the cardiac cycle, (2) its user-friendly interface and (3) an increasing number of studies reporting its application in different clinical settings. In addition, the good agreement between EBD and FT has implications for the clinician, as the adequacy of tracking the endocardial border by the FT software is easily verifiable visually by the user. As a consequence, if the automatic border tracking appears visually adequate, the results of the analysis for global strain are credible. There is no such simple approach to assess the accuracy of segmental strain.

### Right ventricular function

Consistent with previous studies RV function was found to be reduced in ToF patients when compared to controls [12,36]. This is likely due to the combined effects of early pressure overload and hypoxia prior to the corrective surgery and volume overload due to pulmonary regurgitation in later life [37]. Our data specifically support an association between impairment of RV function and the number of previous cardiac surgeries. This topic has been subject to numerous studies over the last decades [38-40]. In most of these studies RV function has been assessed using echocardiographic tricuspid annular plane systolic excursion (TAPSE) and parameters derived from tissue Doppler echocardiography (TDI). These parameters are, however, angle dependent, influenced by tethering and therefore do not directly reflect myocardial function. CMR based assessment of the RV - employing FT, as used in the current study - enables measuring myocardial deformation parameters in 3 orthogonal planes in addition to conventional volumetric parameters of RV function. The current study shows that longitudinal strain and ejection fraction are impaired decades after cardiac surgery and there seems to be a cumulative effect of cardiac surgery on RV impairment, with a negative correlation between number of surgeries and RV function.

**Figure 11:**
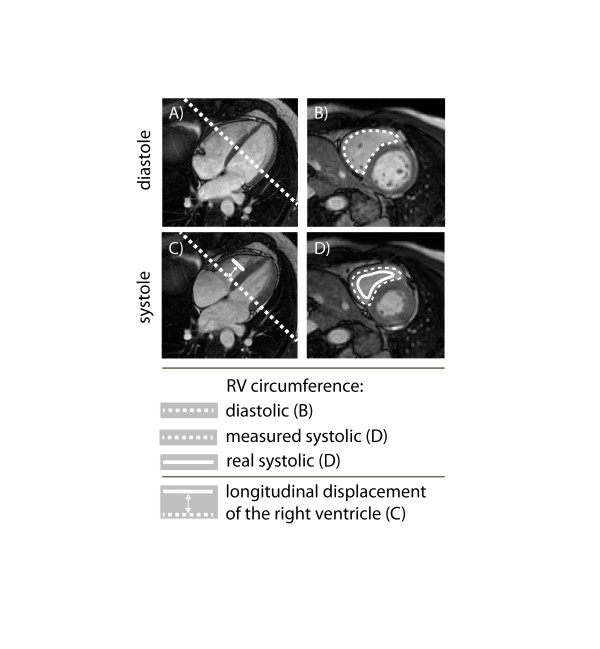
**Example of out of plane motion of the region of interest.** Diastolic circumference of the RV (L_0_) is obtained on the mid-ventricular level (B). Since the scanning plane doesn’t move, while heart tissue is displaced apically during contraction, the systolic circumference is obtained (D) from an initially more basal located RV level. Absolute circumferential Lagrangian strain (CS) is based on the circumference measurements and calculated as CS = 100%*(diastolic-systolic)/diastolic. Therefore, the CS values based on measured systolic circumference are falsely reduced. This effect is exaggerated with higher longitudinal displacement (as is the case in healthy controls compared to patients with tetralogy of Fallot).

Interestingly, circumferential RV strain was found to be higher in ToF patients compared to controls and there was also a similar trend for the radial RV strain. This finding has not been reported in previous studies employing echocardiographic methods, since measurement of these parameters is not possible in the majority of ToF patients on echocardiography due to suboptimal acoustic window. The reason for increased circumferential strain in ToF patients remains unclear. As control subjects had higher longitudinal strain values, this may lead to more pronounced out of plane motion and thereby contribute to underestimation of circumferential strain as illustrated in Figure 11. Therefore, the 2D circumferential and radial RV strain assessed in this study appears to be a combined parameter reflecting both longitudinal, circumferential and radial RV deformation. Moreover, the significant relation of the FT circumferential and radial RV strain to exercise capacity illustrates its physiologic relevance. Although the clinical consequences of these findings remain unclear at present, we believe they illustrate the potential of the technology in assessing myocardial performance and putting it into the context of functional capacity. As parameters of CPET are, in themselves, related to prognosis in ACHD, further studies assessing the predictive value of RV strain in this setting are warranted [41].

### Clinical implications

Measurement of myocardial deformation using FT is feasible in a clinical setting and shows good agreement with measurements obtained from STE. As parameters of myocardial deformation are thought to better reflect early myocardial dysfunction than volumetric parameters they may have the potential to improve prognostication and guide treatment in the setting of ToF. In addition, unlike ejection fraction RV strain measurements were related to exercise capacity in this population, supporting its clinical usefulness. Unlike CMR tagging techniques, FT does not require special acquisition protocols and can also be used to evaluate routine CMR data. We suggest that FT derived measures of longitudinal and radial strain should be considered as adjuncts to conventional volumetric parameters of RV and LV function in ToF patients for studies assessing the prognostic value and the impact of interventions/surgery on biventricular function in this setting.

Strain assessment on FT can be performed using commercially available software that does not require extensive training. The analysis is not more labour intensive than conventional STE (requiring only approx. 5 minutes) and FT strain measurements can be obtained in a higher proportion of patients than STE.

### Limitations

Right ventricular strains reported in this study reflect the function of the basal and middle part of RV but not the outflow, where significant dysfunction on other techniques has been observed [36]. Three-dimensional feature tracking with assessment of outflow tract would be more optimal in this setting and would also eliminate errors resulting from out of plane motion. Measuring RV outflow tract function is however not possible with the FT software, as currently implemented. Further research is required to elucidate the reasons between the discrepancy of good reproducibility of global strain and FT problems in reproducing regional strain measurements.

RV EF enables in this setting a more comprehensive evaluation and reflects also RVOT contractility.

The frame rate on CMR in our study was lower than that employed in many software solutions for STE. The TomTec FT software was, however, developed for CMR use and the frame rate in our study (25-33 Hz) is identical to that reported in other CMR studies assessing myocardial strain [23,24]. In addition, we focused on global systolic strain, which should be less affected by the temporal resolution compared to parameters of diastolic function or measures of asynchrony.

We have not assessed in the current study either inter-study repeatability or accuracy of any of the measurements of strain. Further studies are required to assess this.

Due to the limited number of ToF patients with different degrees of pulmonary regurgitation included in the study we are unable to draw conclusions on the association between severity of PR and strain measures on FT. Further studies, including a larger number of patients are required to address this issue.

## Conclusion

Feature tracking provides measurements of RV global strain in 3 orthogonal planes in patients with tetralogy of Fallot, potentially providing additional insights into the complex function of the RV and assessment of myocardial status. Assessment of LV longitudinal global strain is feasible using FT and STE. However, the results of this study suggest that FT may be superior to STE in assessing RV longitudinal global strain due to its higher inter-observer reproducibility and the possibility to assess RV strain in a higher proportion of patients. In addition, RV radial and circumferential global strain can be measured exclusively on FT. In contrast, reproducibility of segmental strain on FT was poor in our hands and this aspect requires further study. Using EBD, the current study illustrates that dedicated FT software is not necessarily required to obtain reproducible global circumferential endocardial strain measurements, showing agreement with those derived from FT analysis. Clinically, parameters of RV function derived from FT seem to relate more closely with exercise tolerance than conventional volumetric RV functional parameters. Larger studies are needed to assess the relation of this technique to the outcome in ToF patients.

## Misc

This study was supported by a research grant from the EMAH Stiftung Karla Voellm, Krefeld, Germany. AK was supported by the Deutsche Herzstiftung.

## Competing interest

The authors declare that they have no competing interest.

## Authors’ contributions

AK, SO, RF-J, PS, ACB, DM and G-PD collected and analyzed the data. AK and GD wrote the EBD codes and analysed the data using this method. AK, G-Dand HB have written the manuscript. All authors read and approved the final manuscript.

## Supplementary Material

Additional file 1 **Description of additional data files.** Example of feature tracking analysis using TomTec Diogenes software (TomTec, Unterschleissheim, Germany) in a patient with repaired tetralogy of Fallot. Click here for file

Additional file 2 **Description of additional data files.** Example of feature tracking analysis using TomTec Diogenes software (TomTec, Unterschleissheim, Germany) in a patient with repaired tetralogy of Fallot. Click here for file

Additional file 3 **Description of additional data files.** Example of feature tracking analysis using TomTec Diogenes software (TomTec, Unterschleissheim, Germany) in a patient with repaired tetralogy of Fallot. Click here for file

Additional file 4 **Description of additional data files.** Example of feature tracking analysis using TomTec Diogenes software (TomTec, Unterschleissheim, Germany) in a patient with repaired tetralogy of Fallot. Click here for file
